# Defining the Antigenic Diversity of *Plasmodium falciparum* Apical Membrane Antigen 1 and the Requirements for a Multi-Allele Vaccine against Malaria

**DOI:** 10.1371/journal.pone.0051023

**Published:** 2012-12-05

**Authors:** Damien R. Drew, Anthony N. Hodder, Danny W. Wilson, Michael Foley, Ivo Mueller, Peter M. Siba, Arlene E. Dent, Alan F. Cowman, James G. Beeson

**Affiliations:** 1 The Burnet Institute for Medical Research and Public Health, Melbourne, Australia; 2 The Walter and Eliza Hall Institute of Medical Research, Parkville, Australia; 3 La Trobe University, Bundoora, Australia; 4 Papua New Guinea Institute of Medical Research, Goroka, Papua New Guinea; 5 Centre for Global Health and Diseases, Case Western Reserve University, Cleveland, Ohio, United States of America; 6 Department of Microbiology, Monash University, Victoria, Australia; University of Copenhagen, Denmark

## Abstract

Apical Membrane Antigen 1 (AMA1) is a leading malaria vaccine candidate and a target of naturally-acquired human immunity. *Plasmodium falciparum* AMA1 is polymorphic and in vaccine trials it induces strain-specific protection. This antigenic diversity is a major roadblock to development of AMA1 as a malaria vaccine and understanding how to overcome it is essential. To assess how AMA1 antigenic diversity limits cross-strain growth inhibition, we assembled a panel of 18 different *P. falciparum* isolates which are broadly representative of global AMA1 sequence diversity. Antibodies raised against four well studied AMA1 alleles (W2Mef, 3D7, HB3 and FVO) were tested for growth inhibition of the 18 different *P. falciparum* isolates in growth inhibition assays (GIA). All antibodies demonstrated substantial cross-inhibitory activity against different isolates and a mixture of the four different AMA1 antibodies inhibited all 18 isolates tested, suggesting significant antigenic overlap between AMA1 alleles and limited antigenic diversity of AMA1. Cross-strain inhibition by antibodies was only moderately and inconsistently correlated with the level of sequence diversity between AMA1 alleles, suggesting that sequence differences are not a strong predictor of antigenic differences or the cross-inhibitory activity of anti-allele antibodies. The importance of the highly polymorphic C1-L region for inhibitory antibodies and potential vaccine escape was assessed by generating novel transgenic *P. falciparum* lines for testing in GIA. While the polymorphic C1-L epitope was identified as a significant target of some growth-inhibitory antibodies, these antibodies only constituted a minor proportion of the total inhibitory antibody repertoire, suggesting that the antigenic diversity of inhibitory epitopes is limited. Our findings support the concept that a multi-allele AMA1 vaccine would give broad coverage against the diversity of AMA1 alleles and establish new tools to define polymorphisms important for vaccine escape.

## Introduction

There is strong need for vaccines against malaria to combat the global burden of disease, particularly in light of increasing drug resistance [1] and the declining efficacy of vector control interventions [2]. Apical Membrane Antigen 1 (AMA1) is a leading vaccine candidate that is expressed by merozoites of *Plasmodium falciparum* [3], [4]. AMA1 is essential for blood-stage replication [5] and antibodies to AMA1 inhibit erythrocyte invasion [6], [7] making AMA1 an attractive candidate on biological grounds. AMA1 vaccines have demonstrated protective efficacy in rodent and simian models, and evidence suggests that AMA1 is an important target of naturally-acquired protective antibodies in humans [6]–[10]. Antigenic diversity in AMA1 can mediate immune escape and immunisation with AMA1 in animal models is typically protective against homologous parasite challenge with reduced protection against heterologous strains [11]–[14]. The capacity of AMA1 to elicit protective immunity in humans was recently demonstrated by a phase 2b vaccine trial of 1–6 year old children in Mali [15]. This trial, using a vaccine based on a single AMA1 allele, reported 64% protective efficacy against malaria episodes caused by vaccine-like alleles, but no significant efficacy against episodes due to other alleles. These findings demonstrate the potential of AMA1-based malaria vaccines, but highlight the need for strategies to overcome antigenic diversity and prevent vaccine escape.

Currently, there is a limited understanding of antigenic diversity of AMA1 and the requirements of AMA1 vaccines to give broad population coverage. Approximately 10% of the amino acid residues in AMA1 are polymorphic [16] and over 200 unique haplotypes of AMA1 have been identified [17]–[18]. Despite this diversity, recent sequence-based analyses suggests that sequences may be grouped into as few as six clusters of related sequences [18], although others suggest the number might be much larger [17] [19]. It is unclear how sequence polymorphisms and sequence-based groupings relate to antigenic diversity and escape from inhibitory antibodies. Studies of the antigenic diversity of inhibitory epitopes have generally been limited to testing small numbers of *Plasmodium falciparum* laboratory strains and provide limited insight into the number and type of AMA1 alleles required for an effective vaccine.

Clusters of polymorphisms that might contribute to antibody escape have been identified on all three domains of AMA1 [20], although domain 1 appears to be the major target of inhibitory antibodies [21]. One cluster, known as C1-L, spans amino acids 196 to 207 of domain 1. The invasion-inhibitory monoclonal antibody 1F9 binds this region, [22] and studies using recombinant AMA1 proteins suggest this region is an important target of strain-specific inhibitory antibodies. C1-L contains among the most polymorphic residues of AMA1, including the heptamorphic position 197. Longitudinal studies have associated polymorphisms in C1-L with the development of clinical malaria, suggesting it is a target of naturally acquired strain-specific AMA1 immunity [17].

In this study, we aimed to address these gaps in our knowledge and advance the development of a broadly effective AMA1 vaccine. We compiled a diverse array of *P. falciparum* isolates to i) measure the cross-inhibitory capacity of antibodies to different AMA1 alleles generated by immunisation, ii) understand antigenic diversity of inhibitory epitopes, iii) assess relationships between sequence polymorphisms and immune escape, and iv) investigate whether a multi-allele vaccine may be an effective and feasible strategy to overcome antigenic diversity. Our studies focussed on the growth-inhibitory activity of AMA1 antibodies because this is thought to be the major mechanism of action [7]. Furthermore, we developed a novel approach using transgenic *P. falciparum* with defined mutations in AMA1 sequences to identify key polymorphisms, or polymorphic regions, responsible for escape from growth-inhibitory antibodies. Using this approach, we quantified the importance of residues in the C1-L for antibody escape.

## Methods

### Expression of Recombinant AMA1

The DNA sequences encoding W2Mef, 3D7, HB3 and FVO a*ma*1 alleles were codon optimised for expression in *E.coli* (Genscript). The DNA sequence encoding amino acids 25 to 546 of the codon optimised ectodomain was PCR amplified using the oligonucleotide primer sets A-D (Table 1) and ligated into the 6×His expression vector pProEX HTb (Invitrogen) using *Bam* H1 and *Xho* 1 restriction sites. Plasmids were transfected into BL21 *E.coli.* Recombinant AMA1 expression, purification and refolding was performed as previously described [6]. In brief, all proteins were expressed as insoluble inclusion bodies in *E. coli* and then solubilised in 6M Guanidine-HCL, which completely denatures the recombinant proteins. After purification on nickel resin, AMA1 protein was refolded with reduced and oxidized glutathione redox pairs. Refolded AMA1 was further purified by anion-exchange chromatography, followed by reverse phase high-performance liquid chromatography. Refolded AMA1 was identified by a shift in the monomer peak on reverse phase HPLC and a migration shift on SDS-PAGE when compared to a reduced and alkylated sample of the refolded AMA1 preparation.

**Table 1:** Oligonucleotides used for amplification of *ama1* gene sequences.

*Set*	*Oligonucleotide*	*5′ to 3′ DNA sequence*
A	W2EctoCOFP.1	CATGGGATCCCAGAATTACTGGGAACACCCGTATCAG
A	W2EctoCORP.1	GCATGCCTCGAGACTGCAGTCATTTCATTTTATCGTAGGTCGGTTTGTGCTCAGGG
B	3D7EctoCOFP.1	CATGGGATCCCAGAACTACTGGGAACATCCGTATCAGAACTCAGACG
B	3D7EctoCORP.1	GCATGCCTCGAGACTGCAGTCATTTCATCTTGTCATACGTCGGTTTATGTTCAGG
C	HB3EctoCOFP.1	CATGGGATCCCAAAATTACTGGGAACATCCGTATCAGAACAGTGATG
C	HB3EctoCORP.1	GCATGCCTCGAGTCATTTCATATTATCATAGGTTGGCTTGTGTTCCGGG
D	FVOEctoCOFP.1	CATGGGATCCCAGAACTATTGGGAGCACCCATATCAGAAAAGCGACG
D	FVOEctoCORP.1	GCATGCCTCGAGTCATTTCATATTATCGTAGGTTGGTTTATGTTCCGG
E	W2AMA1TFP.1	GGACTAGTGGAAGAGGACAGAATTATTGGGAACATCCATATC
E	W2AMA1TRP.1	CCGCTCGAGTCAACAATTTCCATCGACCCATAATCCGAATTTTGC
F	W2AMA1PFP.1	CCGCTCGAGATTAATGAGGTGTGTTGGGAAACAGAAGAAAAAAAC
F	W2AMA1PRP.1	CGGGGTACCTTGAAAAATTATCAAATAAATTATTAGGTTTTTAAAATTA
G	PfAMA1UnistrainFp.1	GAGAAAATTATACTGCGTATTATTATTGAGCGCCTTTGAG
G	PfAMA1UnistrainRp.1	GCTGAAAAATATGATAAAATGGATGAACCACAAGATTATGGG
H	3D7AMACOKpnFMP.1	AAAGTCGGTACCATTAATGTACTTGTTATAAATTGTACAAAAATGAGAAAATTATACTGCG
H	3D7AMACOPstRMP.1	CATGCTGCAGACTAGTTCAGTAATAAGGTTTTTCCATCAGAACCGGTGTGGTATGGGACGC
I	3D7-GD1FMP.1	CTGAACGGTATGCGTGACTTCTACAAAAACAATGAATACGTGAAGAATCTGGATGAGCTG
I	3D7-GD1RMP.1	CGTATTCATTGTTTTTGTAGAAGTCACGCATACCGTTCAGAGTCATCGGCGACATCAGAGG
J	FVOAMACOKpnFMP.1	AAAGTCGGTACCATTAATGTACTTGTTATAAATTGTACAAAAATGAGAAAATTATACTGCG
J	FVOAMACOPstRMP.1	CATGCTGCAGACTAGTTTAATAATATGGTTTTTCCATCAGCACAGGCGTAGTGTGACTCGC
K	FVO-EH1FMP.1	CCCTGGACGAGATGCGTCACTTTTATAAAGACAATAAATACGTTAAAAACCTGGATGAACTG
K	FVO-EH1RMP.1	CGTATTTATTGTCTTTATAAAAGTGACGCATCTCGTCCAGGGTCATCGGAGAAATCAGCGGG
L	W2AMACOKpnFMP.1	AAAGTCGGTACCATTAATGTACTTGTTATAAATTGTACAAAAATGAGAAAATTATACTGCG
L	W2AMACOPstRMP.1	CATGCTGCAGACTAGTTCAATAATACGGCTTTTCCATCAGAACCGGGGTGGTATGACTTGC
M	W2-EH1FMP.1	CTGGATGAGATGCGTCACTTCTACAAAGACAACAAAGACGTGAAAAATCTGGATGAACTGACC
M	W2-EH1RMP.1	GTCTTTGTTGTCTTTGTAGAAGTGACGCATCTCATCCAGCGTCATAGGAGACATCAGCGG
N	W2-QH1FMP.1	CTGGATCAGATGCGTCACCTGTACAAAGACAACGAAGACGTGAAAAATCTGGATGAACTGACC
N	W2-QH1RMP.1	GTCTTCGTTGTCTTTGTACAGGTGACGCATCTGATCCAGCGTCATAGGAGACATCAGCGG
O	W2-GD1FMP.1	CTGAATGGTATGCGTGACTTCTACAAAAACAACGAAGACGTGAAAAATCTGGATGAACTGACC
O	W2-GD1RMP.1	GTCTTCGTTGTTTTTGTAGAAGTCACGCATACCATTCAGCGTCATAGGAGACATCAGCGG
P	W2-DR1FMAP.1	CTGGATGATATGCGTCGTTTCTACAAAGACAACGAAGACGTGAAAAATCTGGATGAACTGACC
P	W2-DR1RMP.1	GTCTTCGTTGTCTTTGTAGAAACGACGCATATCATCCAGCGTCATAGGAGACATCAGCGG
Q	W2-QD1FMP.1	CTGGATCAGATGCGTGACTTCTACAAAAACAACGAAGACGTGAAAAATCTGGATGAACTGACC
Q	W2-QD1RMP.1	GTCTTCGTTGTTTTTGTAGAAGTCACGCATCTGATCCAGCGTCATAGGAGACATCAGCGG
R	W2-DN1FMP.1	CTGGACGACATGCGTAACTTCTACAAAGACAATGAAGACGTGAAAAATCTGGATGAACTGACC
R	W2-DN1RMP.1	GTCTTCATTGTCTTTGTAGAAGTTACGCATGTCGTCCAGCGTCATAGGAGACATCAGCGG

### Immunisation of Rabbits and Generation of Antibodies

New Zealand White rabbits were immunised with 50 µg of recombinant AMA1 emulsified in Freunds adjuvant. Rabbits were injected intramuscularly three times over 4 week intervals using standard procedures. Ethics clearance for rabbit immunisations performed in this study was obtained from the Animal Ethics Committee of the Walter and Eliza Hall Institute, Australia. Sera was heat inactivated and screened for growth inhibition by GIA. Sera generating the highest level of growth inhibition against homologous isolates were selected for use in cross-strain GIAs (anti-W2Mef #1, anti-3D7#1, anti-HB3#1 and anti-FVO#1). Anti-3D7, anti-HB3 and anti-FVO AMA1 antibodies were adjusted to 20 mg/ml of total IgG after purification on protein-G sepharose. Anti-W2Mef #1and anti-FVO#2 rabbit antisera were generated as previously described and tested as whole serum in GIAs [6]. A pool of anti-W2Mef #1, anti-3D7#1, anti-HB3#1 and anti-FVO#1 antibodies was generated by combining equal volumes of each of the four antibodies.

### Parasite Culture and Genotyping

*P. falciparum* asexual stage parasites were maintained in culture in human erythrocytes (blood group type O+) at a hematocrit of 4% in RPMI-HEPES supplemented with 0.25% (w/v) Albumax™ (Invitrogen) and 5% (v/v) heat inactivated human serum [23]. The origins of *P. falciparum* isolates used in this study are listed in Table 2. [24]–[35] Isolates XHA-A, XHA-D, and BFD06 are described for the first time in this study. Isolates XHA-A and XHA-D were derived from the peripheral blood of two different children in PNG (Madang Province, year 2004) [36] [10] and adapted to in vitro culture over several weeks. BFD06 was isolated in 2006 from an adult with acute malaria who returned from travelling in Burkina Faso. The recovered parasites were restrictively diluted to isolate pure strains with isolate “D” perpetuated in culture. Genomic DNA was prepared from each isolate, the sequence encoding the AMA1 ectodomain (aa 25–546) was amplified by PCR and directly DNA sequenced using oligonucleotide set G (Table 1).

**Table 2:** *P. falciparum* isolates used in this study.

*Isolate*	*Origin*	*Reference*
W2Mef	SE Asia	[24]
3D7	Amsterdam Airport, origin unknown	[25]
HB3	Honduras	[26]
FVO	Vietnam	[28]
7G8	Peru	[29]
XIE	PNG	[30]
Pf2004	Ghana	[31]
Pf2006	Ghana	[31]
Camp	Malaysia	[32]
D10	PNG	[27]
E8B	Unknown (derived from IT4)	[33]
HCS-E5	Thailand	[30]
K1	Thailand	[34]
CSL-2	SE Asia	[35]
XHA-A	PNG	Unpublished
XHA-D	PNG	Unpublished
T996	Thailand	[27]
BFD06	Burkina Faso	Unpublished

### *P. falciparum* Growth Inhibition Assays

*P. falciparum* growth inhibition assays (GIA) were performed as described [37]–[40] with the following modifications: Synchronised early trophozoite stage parasites were adjusted to 0.1% parasitemia and 2% hematocrit. Five µl of stock antibody or peptide was added to 45 µl of infected red blood cells and mixed to generate a final culture volume of 50 µl. Parasites were allowed to develop through two cycles of erythrocyte invasion for 72 hours at 37°C. Early trophozoite stage parasites formed after the second round of invasion were fixed for 1 hour at room temperature by the addition of Glutaraldehyde (ProSciTech) to a final concentration of 0.25% (v/v). After fixation, parasites were washed in HTPBS, stained with 10× SYBR green dye (Invitrogen) and 5×10^5^ red blood cells counted per well using a BD FACSCantoII flow-cytometer. FACS counts were analysed using FlowJo™(Ver 6.4.7) software (Treestar). Percent growth inhibition = (1−[parasitemia in test well/mean parasitemia in pre-immunization rabbit controls wells])×100. All GIAs were run in a 96-well plate format, with each antibody or peptide tested in triplicate wells. Parasite growth inhibition is represented as the combined mean of two separate triplicate well assays set up on different days. Growth inhibition by rabbit antibodies is expressed relative to non-immunized control rabbits; there was no significant inhibition of growth by IgG or whole serum from non-immunized control rabbits. Purified antibodies were tested at a final concentration of 2 mg/ml total IgG; normal physiologic concentration of total IgG is 10–15 mg/ml. Since it is not known how the levels of inhibition in GIAs in vitro relate to the inhibitory or protective activity of antibodies in vivo, the levels of inhibition by individual antibodies and antibody pools tested in GIAs should be regarded as relative inhibition, rather than absolute levels of inhibition, allowing comparisons of the inhibitory activity between antibodies and isolates. For analysis of the relationship between antibody inhibition and polymorphisms in AMA1, results were standardized such that the level of inhibition of the homologous isolate by anti-allele antibodies was designated as 100% (E.g. Inhibition of 3D7 parasites by anti-3D7 antibodies was designated as 100%; no inhibition was 0%). This was done to facilitate comparisons of the inhibitory effects of different antibodies against the panel of different isolates; the standardisation did not affect the correlation coefficients or p values.

### Generation of Transgenic *P. falciparum*

Transfection plasmids were assembled in pCC1 [41]. This vector contains a hDHFR cassette driven by the Calmodulin promoter. Expression of hDHFR mediates resistance to drug WR99210 and enables positive selection of transfected parasites. pCC1 also contains a ganciclovir resistance CD cassette which was removed and replaced by an *ama1* allelic exchange cassette consisting of a W2Mef AMA1 targeting domain, the W2Mef AMA1 promoter and an *E.coli* codon-optimised form of the new AMA1 allele to be introduced. The W2Mef AMA1 targeting domain was amplified by PCR using the oligonucleotide primer set E (Table 1) and inserted into pCC1 using Spe1 and Xho1 restriction sites. This 917bp PCR product consists of the 5′ domain of W2Mef AMA1 with a stop codon engineered at its 3′ end. The 1429 bp W2Mef AMA1 promoter was PCR-amplified using the oligonucleotide primer set F (Table 1) and inserted into pCC1 using Xho1 and Kpn1 restriction sites to create the plasmid pCC1AMA1TP.1. Codon-optimised W2Mef, 3D7 and FVO AMA1 alleles were synthesized with a 30 bp 5′ UTR tag ahead of the ATG start codon, in order to maintain the W2Mef AMA1 kozac consensus sequence upon integration. The 3F3 and F3F hybrid C1-L alleles were constructed by Spliced Overlapping End (SOE) PCR, using codon-optimised 3D7 or FVO AMA1 DNA as templates and oligonucleotide sets H, I, J and K respectively (Table 1). W2Mef hybrid alleles containing the C1-L sequence of 3D7, HB3, FVO, XIE, 2006 and *Plasmodium reichenowi* C1-L (aa196–206) were constructed by SOE PCR using a codon-optimised W2Mef AMA1 template, flanking oligonucleotide set L and internal oligonucleotide sets M to R respectively (Table 1). All AMA1 alleles were inserted into pCC1TP.1 using *Kpn* 1 and *Pst* 1 restriction sites. 3D7 and W2Mef parental parasites were transfected using standard protocols [42]. Transfected parasites were recovered after positive selection with WR99210 and underwent Southern blot analysis to confirm AMA1 allelic replacement had occurred. Southern blots were performed using the DIG system (Roche) and genomic DNA extracted with the Dneasy Tissue Kit (Qiagen). The DNA probe used for Southern Blots was amplified from W2Mef genomic DNA using primer set E (Table 1). Parasite populations confirmed to have plasmid integration into the AMA1 target were cloned by limiting dilution, and positive populations confirmed by Southern Blot.

### SDS-PAGE (Polyacrylamide Gel Electrophoresis) and Immunoblot Analysis

SDS-PAGE and Immunoblot analysis of parasite proteins was performed as previously described [41]. Synchronised schizont-stage parasite cultures were saponin-lysed, washed in phosphate buffered saline (PBS) and resuspended in non-reducing SDS sample buffer (Invitrogen). Samples were sonicated and heated to 100°C for 5 minutes prior to SDS-PAGE. Proteins were separated on 3–8% Tris-Acetate gels (Invitrogen) and transferred onto nitrocellulose using the iBlot system (Invitrogen) according to standard protocols. Blots were probed with the relevant anti-AMA1 rabbit antiserum (1∶2000). Blots were also probed with a mouse monoclonal antibody generated against the *P. falciparum* HSP-70 protein (1∶2000) as a loading control. Horseradish peroxidase-coupled (HRP) goat anti-rabbit Ig (1∶2000) or sheep anti-mouse Ig (1∶2000) Millipore) were used as secondary antibodies.

### Phylogenetic and Statistical Analysis

Amino acid sequence alignments and relative amino acid identity scores were performed using Genious 5.5.6. software (Biomatters). Phylogenetic trees were generated using ClustalW. Statistical analysis was performed using Prism 5 (Graphpad Software). Correlations between growth inhibition and sequence identity scores were calculated by linear regression. Paired two-tailed t-tests were used for comparisons between the level of inhibitory activity between antibodies or between different isolates.

## Results

### Cross-inhibitory Activity of Antibodies to Different AMA1 Alleles

**Figure 1:**
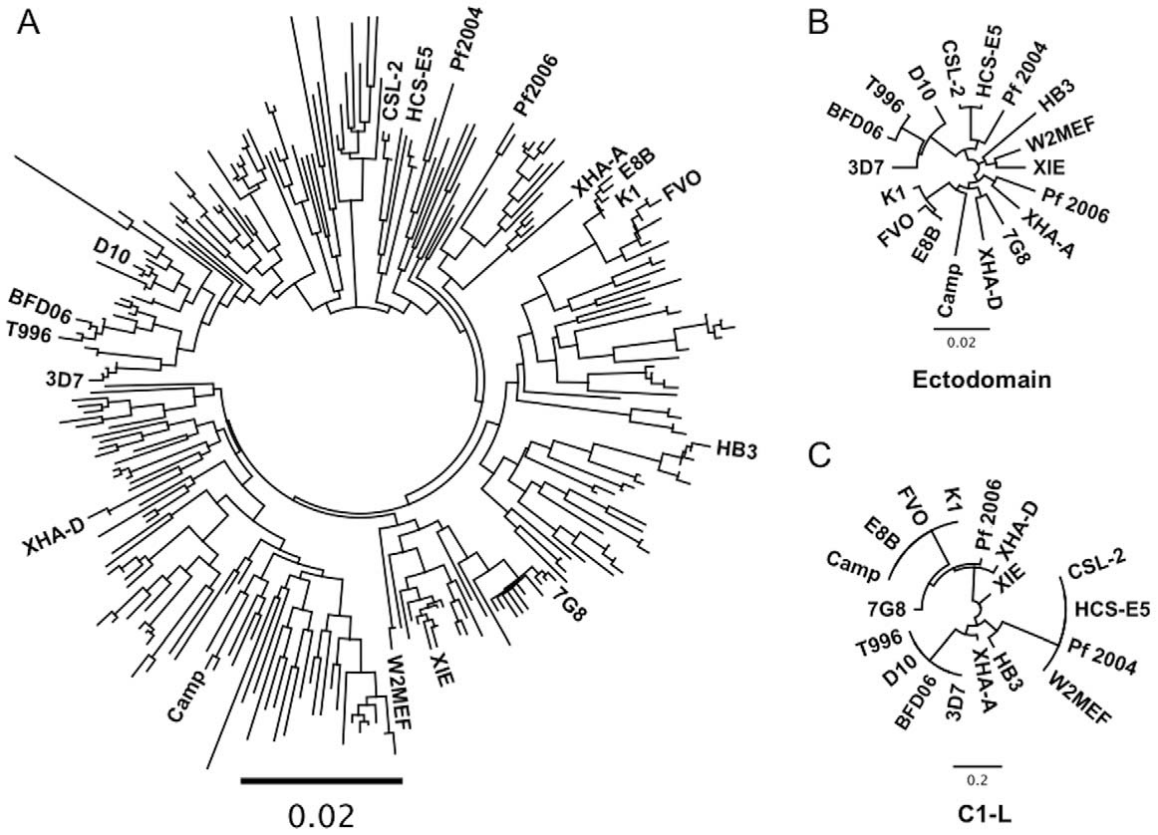
Phylogenetic analysis of AMA1 sequences. **A.** Phylogenetic tree of the AMA1 alleles expressed by 18 different isolates examined in this study in relation to 250 other AMA1 alleles obtained from the public database. Analysis was based on the ectodomain sequence. **B.** Phylogenetic tree of the AMA1 alleles expressed by the 18 different isolates used in this study, based on the AMA1 ectodomain sequence (amino acids 25–456). The AMA1 sequences of HCS-E5 and CSL-2 were found to be identical. **C.** Phylogenetic tree of the AMA1 alleles expressed by isolates used in this study, based on the C1-L sequence of AMA1 (amino acids 196–207). Translated ectodomain and C1-L protein sequences were aligned and phylogenetic trees constructed using ClustalW2.

To understand the antigenic diversity of inhibitory epitopes of AMA1 and the cross-inhibitory activity of antibodies, we generated a panel of 18 different *P. falciparum* isolates that would broadly represent AMA1 global diversity (Table 2). Isolates originated from diverse geographical locations, including Africa, South East Asia, Central and South America and Oceania. AMA1 sequences were determined and all isolates expressed distinct AMA1 alleles except HCS-E5 and CSL-2, which have identical sequences. AMA1 sequences of the isolates were compared to 250 different AMA1 alleles retrieved from the public database (using an NCBI blast search). Analysis showed that the 17 distinct AMA1 alleles were spread throughout a phylogenetic tree (Figure 1A), suggesting they are broadly representative of global AMA1 diversity. Additionally, at least one isolate was included from each of the six haplotype clusters described by Duan *et. al.* 2008 [18]. The four alleles used for antibody generation were selected on the basis that i) they broadly represent global AMA1 diversity based on phyologenetic analysis (Figure 1), ii) they have a varied distribution of polymorphic sites, iii) there is a high level of sequence diversity between the alleles and iv) an isolate corresponding to each allele could be cultured in vitro.

To assess AMA1 diversity within the 17 unique sequences of our 18 isolates, we constructed phylogenetic trees based on either the ectodomain (Figure 1B) or C1-L (Figure 1C). The allelic identity of ectodomain sequences ranged from 100% to 94.5%. Across the 521 aa ectodomain, 61 residues were polymorphic. The vast majority of residues were divalent (52), with amino-acid positions 173, 188, 231, 244 and 504 being trivalent, position 200 quadravalent and position 197 pentavalent. There were nine unique C1-L sequences identified. This diversity is similar to a recent study in Mali which examined 247 AMA1 haplotypes and identified 62 polymorphic residues, of which 46 were divalent [17]. This further indicates that our panel of 18 isolates broadly represents global AMA1 diversity.

**Figure 2:**
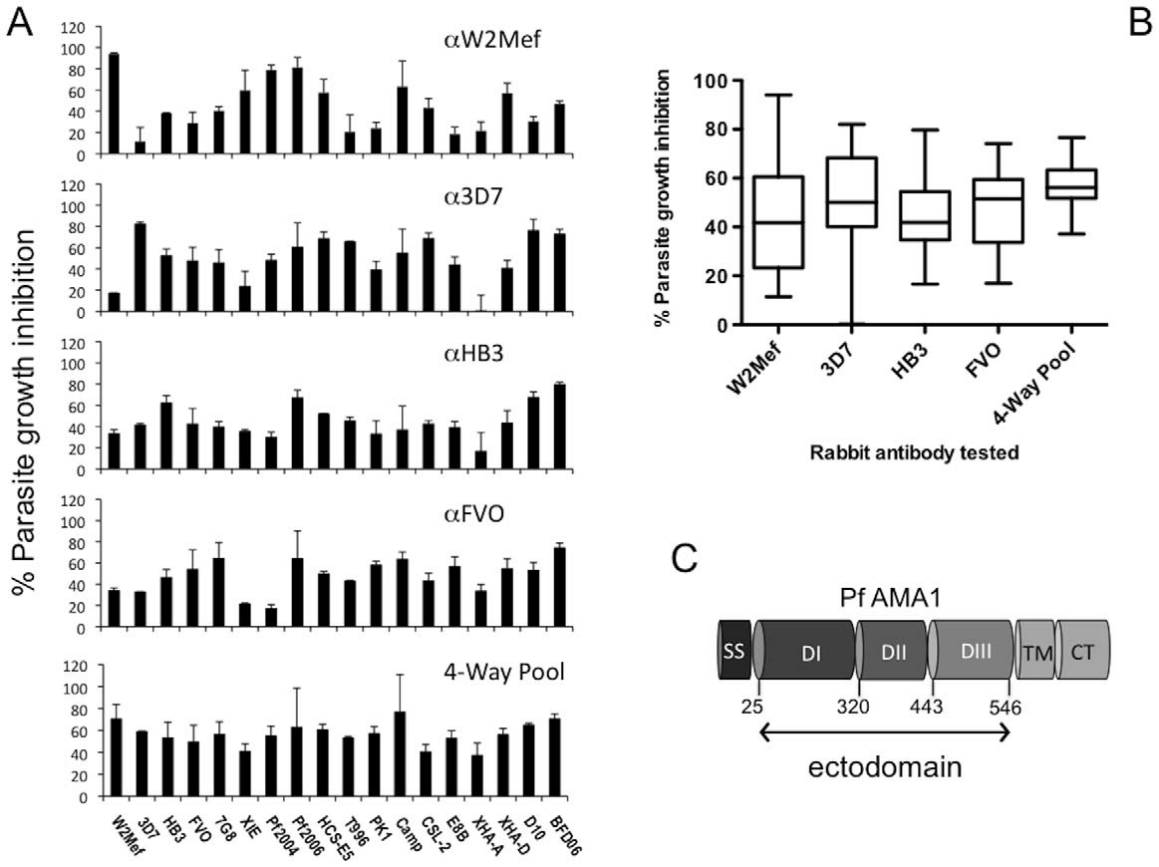
Cross-strain growth inhibition by antibodies to different AMA1 alleles. **A.** The growth-inhibitory activity of polyclonal rabbit antibodies raised against W2Mef, 3D7, HB3 and FVO AMA1 alleles. Anti-W2Mef whole serum was tested at a dilution of 1∶10, and anti-3D7, anti-HB3 and anti-FVO rabbit purified IgG was tested a final concentration of 2 mg/ml IgG. Columns represent the mean parasite growth inhibition achieved in two separate assays tested in triplicate wells. The 4-way pool contains 25% (v/v) of each antibody and was tested at a final dilution of 1∶10. **B** Summary of cross-strain growth-inhibitory activity of antibodies against all isolates. Results show the median (horizontal line) level of inhibitory activity against the 18 isolates tested, and the interquartile range (box) and range (whiskers) of inhibitory activity. **C** Schematic representation of PfAMA1. The positions of amino acids (aa) that define Domain I (D1), Domain II (DII) and Domain III (DIII) of the PfAMA1 ectodomain are shown. The extracellular ectodomain is composed of aa 25 to 546 and excludes the signal sequence (SS), transmembrane domain (TM) and intracellular cytoplasmic tail (CT) regions. Not to scale.

Each of the anti-AMA1 antibodies showed a unique pattern of inhibitory activity against the panel of 18 isolates (Figure 2A). The median level of growth inhibition for all isolates was similar for each of the four anti-AMA1 antibodies (P<0.05), ranging from 42 to 52% (Figure 2B). The maximum level of inhibition achieved by each of the four antibodies ranged between 75 and 95%, while the minimal levels of inhibition ranged between 0 and 17% (Figure 2B). The highest level of inhibition for anti-W2Mef and anti-3D7 antibodies was seen against the corresponding parental isolates. This was not observed for anti-HB3 and anti-FVO antibodies, which showed maximum inhibition against BFD06, a recent African isolate. Several isolates showed low inhibition (≤25%) by individual antibodies, suggesting that they may be candidates for antibody, or vaccine, escape. Of note however, all isolates were inhibited by ≥ 25% by at least one of the 4 antibodies tested. A pool of the four anti-allele antibodies was prepared and tested against all isolates to evaluate the potential for a multi-allele vaccine to give broad inhibitory activity against different isolates. All isolates were substantially inhibited by the antibody pool (Figure 2A). The pool increased the median inhibition across all isolates to 56%, higher than the median inhibition of individual anti-allele antibodies, and no isolate was inhibited by less than 37% (Figure 2B). These findings suggest that antigenic diversity of AMA1 is limited and there is substantial antigenic overlap between different AMA1 alleles. Including four different alleles in a vaccine may be sufficient to overcome AMA1 diversity.

### AMA1 Sequence Diversity and Allele-specific Growth Inhibition

**Figure 3:**
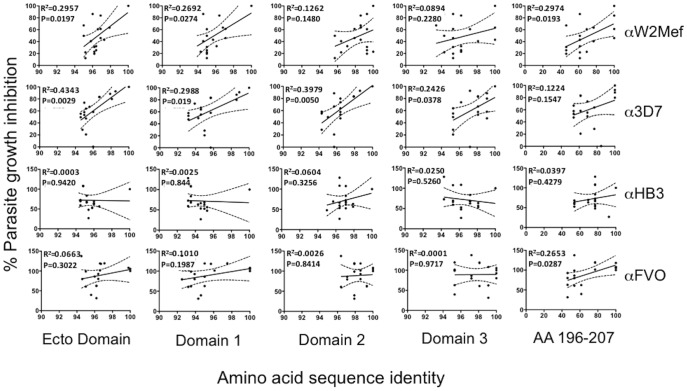
Relationship between sequence polymorphisms and growth inhibition by antibodies. Graphs show regression analysis of normalised percent growth inhibition and AMA1 amino acid (aa) sequence identity. For each antibody, the percent growth inhibition against isolates are plotted against the sequence identity (as a percentage; X axis) relative to the AMA1 allele used to generated the anti-allele antibody tested. This was done for sequences corresponding to the ectodomain (aa 25–546), Domain 1 (aa 25–320), Domain 2 (aa 321–442), Domain 3 (aa 443–546) or C1-L region (aa 196–207). The relationship between AMA1 sequence similarity and growth inhibition was analysed by linear regression. R^2^ and P values are displayed in the top left of each graph. The solid line represents the line of best fit for each scatter plot, and dashed lines represent the standard deviation. Growth inhibition for each isolate by AMA1 antibodies was normalised relative to the inhibition of the parental parasite isolate (e.g.: antibody inhibition of the HB3 isolate by anti-HB3 antibodies was defined as 100%).

We investigated relationships between polymorphisms in AMA1 and levels of growth-inhibitory activity of the different antibodies. AMA1 sequences were aligned and their sequence identity to either W2Mef, 3D7, HB3 or FVO AMA1 was calculated. This was performed for the whole ectodomain, and each of domains 1, 2, and 3 (Figure 2C) and the C1-L polymorphic region. Sequence identity scores were plotted against relative growth inhibition values for each antibody (Figure 3). The level of inhibitory activity by antibodies was not strongly or consistently related to the extent of polymorphism when assessing the entire ectodomain, specific domains or C1-L region. Correlations between sequence identity and inhibition also varied between anti-allele antibodies. Significant correlations, of moderate strength, were seen for the ectodomain and domains 1 for anti-W2Mef and anti-3D7 antibodies, but not for anti-HB3 or anti-FVO. For the C1-L sequence analysis, significant correlations were seen for anti-W2Mef and anti-FVO antibodies, but not antibodies to 3D7 and HB3 alleles. This suggests that the activities of each of the allelic antibodies are influenced differently by polymorphisms across the ectodomain of AMA1. For example, anti-W2Mef and anti-FVO inhibitory antibodies may be more influenced by changes in the C1-L region, whereas anti-3D7 and anti-HB3 inhibitory appear less influenced by this region. However, the overall weak-to-moderate relationships between inhibition and sequence identity highlight the limitations of using sequence analysis, on its own, to define or predict antigenic diversity of inhibitory epitopes.

### Evaluating the Importance of C1-L Residues for Antibody Escape with the 3D7 Allele

**Figure 4:**
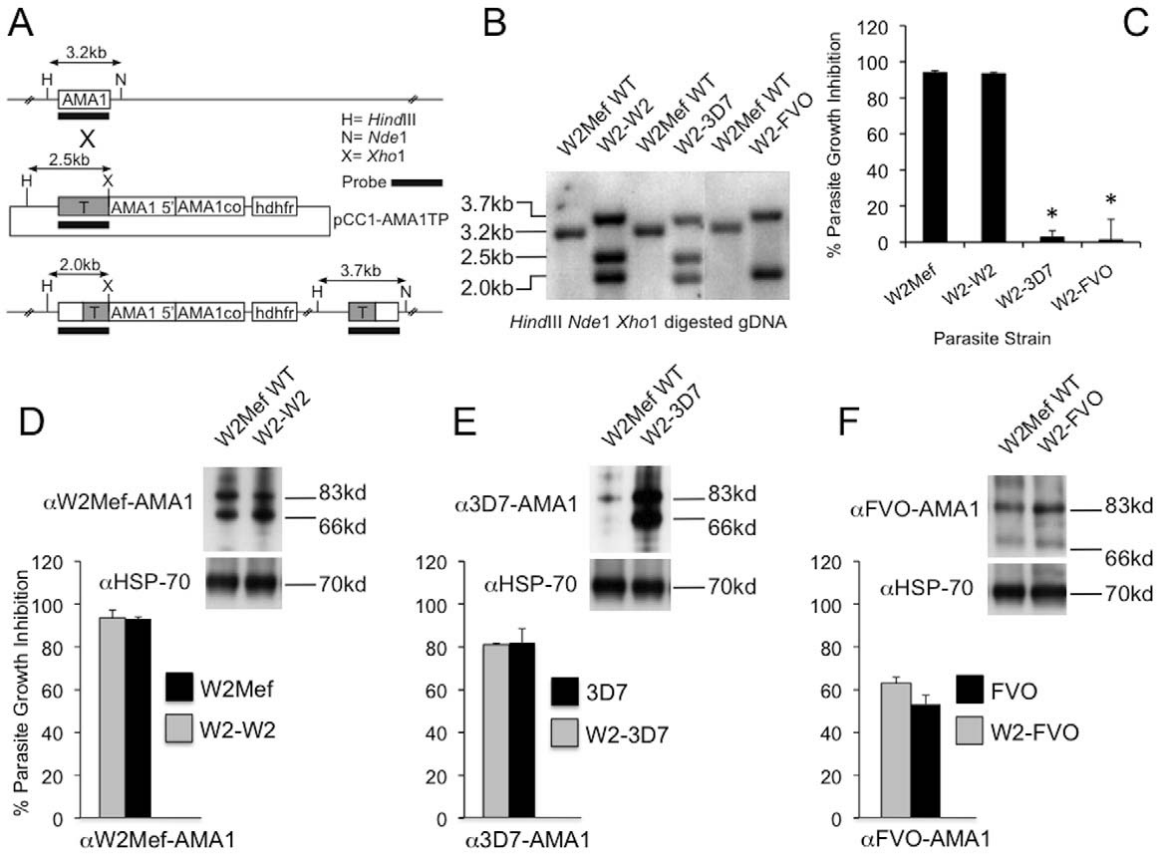
Transgenic and wild type parasite lines expressing the same AMA1 alleles share the same phenotype. A. Plasmid design and integration. Codon optimised W2Mef, 3D7 and FVO AMA1 alleles were transfected into W2Mef parental parasites. The single-crossover event for allelic replacement of the wild type (WT) AMA1 is illustrated. **B.** Southern blot. Genomic DNA from parental W2Mef and transfected parasites was digested with restriction enzymes as indicated and hybridised with an AMA1 probe. Expected sizes for WT, non-integrated plasmid and integrated 3F3-AMA1 are shown in kilobases (kb). **C.** Differential growth inhibition of wild type and transgenic parasite lines by anti-W2Mef #1 antibodies tested at a final dilution of 1∶10. * indicate a significant difference in inhibition when compared to the W2Mef reference line, P<0.05 by t-test. **D, E, F** Phenotypic comparisons between the parental and transgenic parasites for W2Mef, 3D7 and FVO alleles of AMA1. Each figures shows the expression of W2Mef, 3D7 and FVO AMA1 in transgenic parasites compared to the corresponding parental parasite isolate by western blot and growth inhibition of transgenic parasites compared to the corresponding parental parasite isolate with AMA1 allele-specific antibodies.

**Figure 5:**
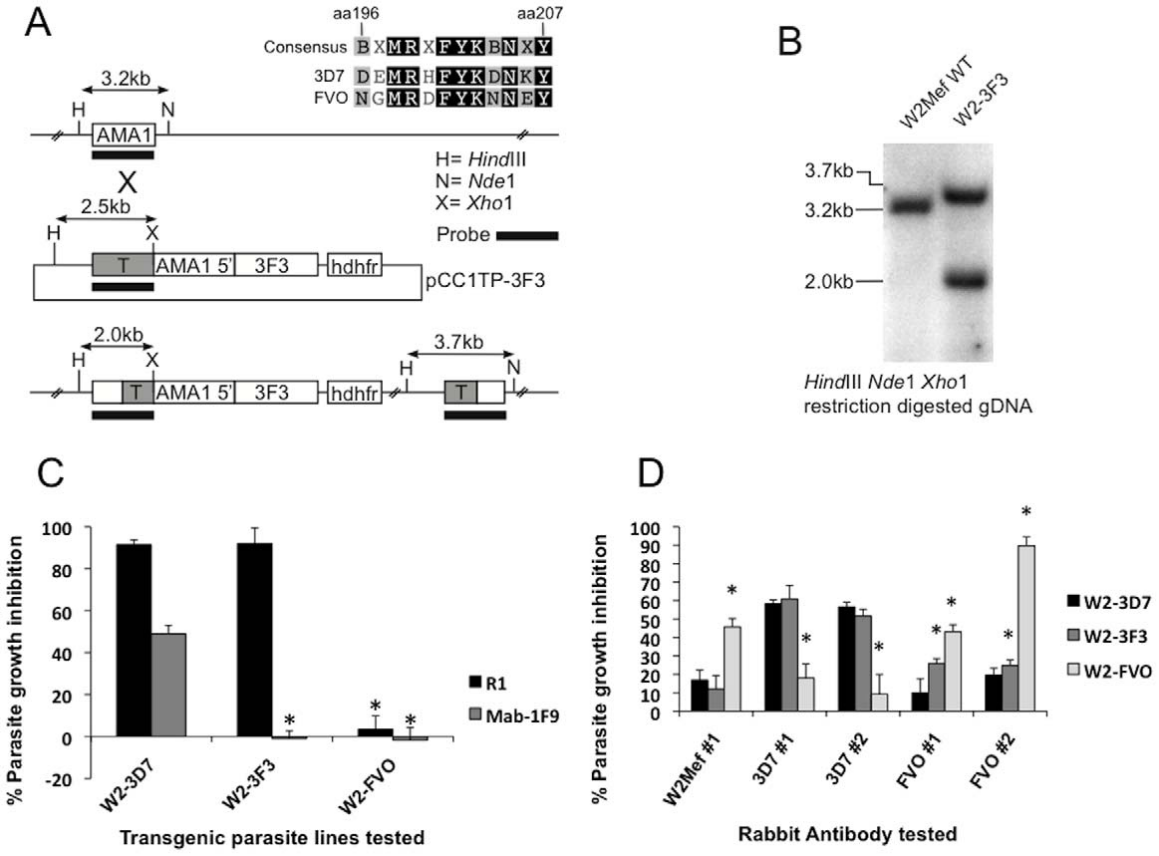
The importance of polymorphisms in the C1-L region of 3D7 for vaccine escape. A. Plasmid design and integration. The C1-L of 3D7 and FVO AMA1 differ by 5 amino acid (aa) residues located at positions 196, 197, 200, 204 and 206. The hybrid 3F3 AMA1 (3D7 allele with the FVO C1-L sequence) was transfected into W2Mef parental parasites. The single-crossover event for allelic replacement of the wild type (WT) AMA1 with 3F3 is illustrated. **B.** Southern blot. Genomic DNA from parental W2Mef and transfected parasites was digested with restriction enzymes as indicated and hybridised with an AMA1 probe. Expected sizes for WT, non-integrated plasmid and integrated 3F3-AMA1 are shown in kilobases (kb). **C.** Phenotypic analysis of transgenic parasites expressing the 3F3-AMA1 hybrid. Transgenic W2Mef parasites expressing 3D7-AMA1 (W2-3D7), FVO-AMA1 (W2-FVO) or the hybrid 3F3-AMA1 (W2-3F3) were tested for their susceptibility to growth inhibition with the R1 peptide (final concentration 100 mg/ml) or the monoclonal antibody 1F9 (final concentration 0.2 mg/ml). **D.** Differential growth inhibition of transgenic parasite lines by polyclonal rabbit antibodies to AMA1; anti-W2Mef #1 and anti-FVO#2 rabbit sera were tested at a final dilution of 1∶10, all other antibodies listed were tested at a final concentration of 2 mg/ml IgG. Columns represent the mean parasite growth inhibition achieved in two separate assays tested in triplicate wells. * indicate a significant difference in inhibition when compared to the W2-3D7 reference line, P<0.05 by t-test.

To dissect the importance of C1-L polymorphisms for escape from growth-inhibitory antibodies, we took a novel approach by generating transgenic parasite lines expressing hybrid AMA1 alleles in which the C1-L region was modified. As controls, we generated W2Mef parasites in which the endogenous AMA1 was replaced with the codon-optimised W2Mef, 3D7 or FVO alleles. Genotypes and phenotypes of transgenic parasites were confirmed by southern blots and Western blots (Figure 4). In GIAs, wild type and transgenic parasites expressing the same AMA1 allele were inhibited at the same level by their corresponding antibodies (Figure 4). In our first model we constructed plasmids containing the 3D7 allele of AMA1 with the C1-L sequence from FVO (named 3F3), or FVO-AMA1 with the C1-L sequence from 3D7 (named F3F). The C1-L region differs by 5 amino acids between 3D7 and FVO at residues 196, 197, 200, 204 and 206 (Figure 5A). Plasmids were transfected into W2Mef parental parasites; however, only the 3F3-*ama*1 gene was successfully integrated into the AMA1 locus (Figure 5B). A second round of F3F transfection was again unsuccessful (data not shown), suggesting that the F3F-AMA1 hybrid allele may not be functional. W2-3F3 parasites completely escaped inhibition by monoclonal antibody 1F9, confirming the loss of the 3D7 C1-L epitope that it binds. On the other hand, R1 peptide inhibits invasion by binding to 3D7-AMA1 independently of the C1-L [43]. W2-3F3 parasites were strongly inhibited by R1, confirming the correct expression of 3F3-AMA1 (Figure 5C). There was minimal inhibition by anti-W2Mef, consistent with the replacement of the W2Mef allele with the 3F3-hybrid allele.

We looked for differential inhibition of the W2-3F3 hybrid compared toW2-3D7 and W2-FVO transgenic parasites in GIAs. For anti-3D7 antibodies, there was no significant difference in inhibition between the W2-3D7 and W2-3F3 transgenic lines (Figure 5D). With anti-FVO antibodies, W2-3F3 parasites showed a slight increase in growth inhibition, compared to that seen for W2-3D7 parasites (FVO #1 P = 0.007, FVO #2 P = 0.024). Given the minimal effect on antibody inhibition of changes in the C1-L region of 3D7-AMA1, mutations in this region were not explored further.

### C1-L Residues and Vaccine Escape with the W2mef Allele

**Figure 6:**
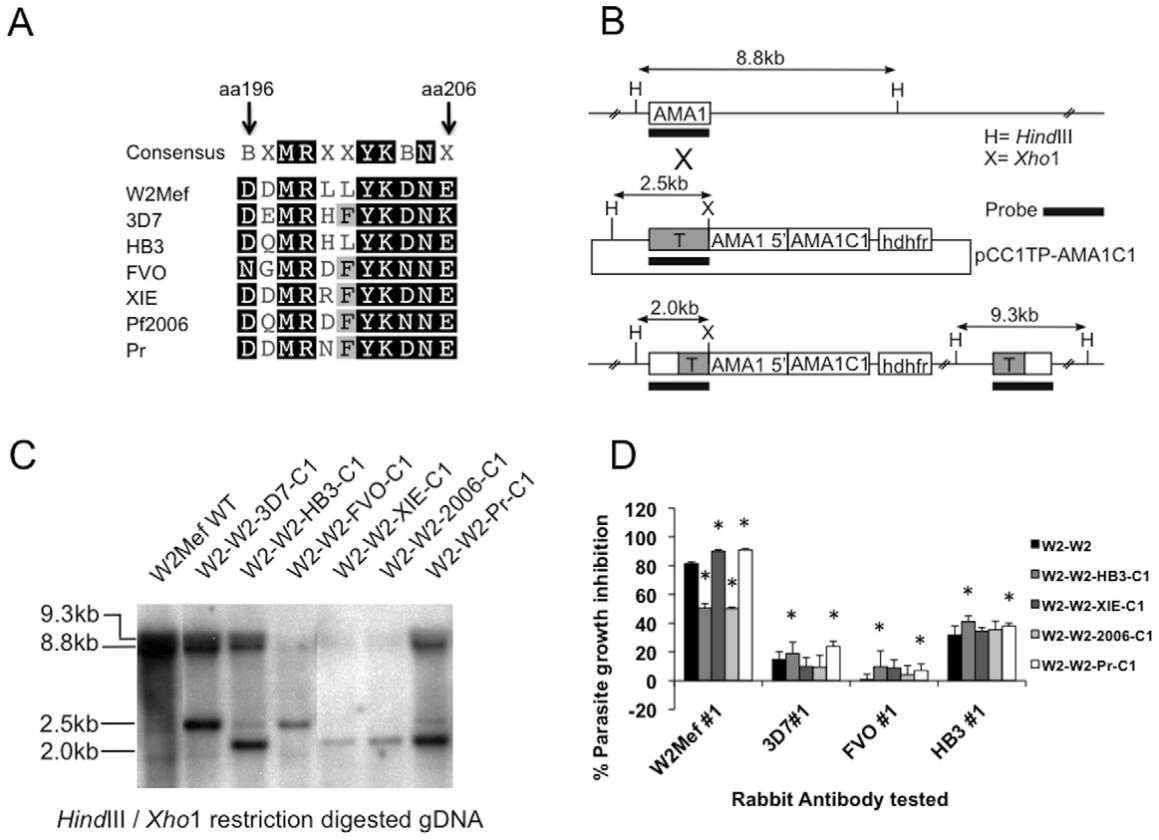
The importance of polymorphisms in the C1-L region of W2mef for vaccine escape. **A** Alignment of amino acids (aa) 196–206 of AMA1 across the different strains. W2Mef, 3D7, HB3, FVO, XIE, Pf2006 and *P. reichenowi* (Pr) AMA1 C1-L 196 to 206 region have polymorphisms at 6 out of the 11 amino acid locations within the defined loop region, located at positions 196, 197, 200, 201, 204 and 206. **B.** Plasmid design and integration. Six hybrid W2Mef-AMA1 alleles containing the aa196–206 C1-L domain from 3D7, HB3, FVO, XIE, Pf2006 or Pr were transfected into W2Mef parental parasites. The single-cross over event for allelic replacement of the wild type (WT) AMA1 with W2Mef-C1-L hybrid AMA1 alleles is illustrated. **C.** Southern blot. Genomic DNA from parental W2Mef and transfected parasite populations was digested with restriction enzymes as indicated and hybridised with an AMA1 probe. Expected sizes for WT, non-integrated plasmid and for integrated W2Mef C1-L hybrids are shown in kilobases (kb). **D** Differential growth inhibition of transgenic parasite lines by polyclonal rabbit antibodies to AMA1. The four W2Mef C1-L hybrid transgenic lines generated, and a control W2–W2 transgenic line expressing WT W2Mef AMA1, were tested in GIAs against different polyclonal rabbit AMA1 antibodies. Anti-W2Mef #1 was tested at a final dilution of 1∶10, and all other antibodies listed were tested at a final concentration of 2 mg/ml of IgG. Columns represent the mean parasite growth inhibition achieved in two separate assays tested in triplicate wells. * indicates a significant difference in inhibition when compared to the W2–W2 reference line, P<0.05 by t-test.

To further assess the importance of C1-L residues, we used a second transgenic model based on W2Mef-AMA1 allele. The significance of C1-L in the W2Mef allele has not been reported, but our preliminary analyses suggested that C1-L polymorphisms may be important. We constructed plasmids containing W2Mef AMA1 with the C1-L from 3D7, HB3, FVO, XIE, or Pf2006 alleles, or from the related species, *P. reichenowi* (Pr), which represented different levels of polymorphism naturally present in populations (Figure 6A). Testing the impact of different modifications on growth inhibition might allow specific residues that are important for vaccine escape to be identified. All plasmids were transfected into W2Mef parental parasites (Figure 6B, 6C). W2Mef hybrid *ama1* genes containing the C1-L sequence of HB3, XIE, Pf2006 or Pr were successfully integrated, whereas the hybrids containing 3D7 or FVO C1-L sequences did not integrate after the first or subsequent transfections. Parasites expressing the HB3 or Pf2006 C1-L sequence were inhibited substantially less than W2Mef (38% lower; p<0.001), whereas parasites expressing the XIE or Pr C1-L did not escape inhibitory activity, and were inhibited by 10% more than W2Mef (P<0.001, Figure 6D). This suggests that specific polymorphisms in the C1-L have a major influence on antibody escape for W2Mef. Both the HB3 and Pf2006 alleles differ from W2Mef at residue 197, whereas XIE and Pr do not. The latter two isolates have polymorphisms at residues 200 and 201, but these did not reduce susceptibility to inhibitory antibodies. Apart from residue 197, the HB3 hybrid allele only differed from W2Mef at residue 200. Together, these findings suggest that changes at residue 197 most strongly influence antibody escape with anti-W2Mef. Of note, the hybrid line expressing HB3 C1-L had inhibition by anti-HB3 antibodies 30% higher that the W2–W2 control (P = 0.0124, Figure 6D), further suggesting the importance of this region as a target of inhibitory antibodies. There was very little inhibition by anti-FVO and anti-3D7 antibodies across the five transgenic lines (Figure 6D).

## Discussion

Our findings provide important new knowledge on AMA1-based vaccines and understanding immune escape. The diversity of AMA1 inhibitory epitopes is lower than expected from sequence analyses, with substantial antigenic overlap between different alleles. Our results support the concept that, when combined, antibodies raised against four different alleles increases median growth inhibition across all isolates. As such, a vaccine containing four AMA1 alleles may cover allelic-diversity, provided the right alleles are selected. Additionally, we developed novel tools and approaches to dissect the importance of specific polymorphic epitopes and quantified the importance of polymorphisms in the C1-L region.

The premise that immune selection drives the evolution of AMA1 diversity suggests that an AMA1 vaccine must encompass this diversity to be broadly cross-protective. Recent studies have attempted to estimate global AMA1 sequence diversity and group AMA1 haplotypes into clusters of related sequences [18], [19] [17], but how this sequence diversity relates to antigenic diversity has been unknown. The results of the present study suggest that antigenic diversity of inhibitory epitopes may be lower than predicted by sequence analyses since a mixture of antibodies to four alleles was sufficient to inhibit a diverse panel of *P. falciparum* isolates. Our findings suggest that sequence differences are not a strong predictor of antigenic differences or the cross-inhibitory activity of anti-allele antibodies. Correlations between the level of sequence polymorphism and cross-inhibitory activity against different isolates were modest and inconsistent across the four anti-allele antibodies, when the whole ectodomain, separate domains or discrete regions were considered. Not all polymorphisms mediate the same level of immune escape (Figures 5 and 6) [44] [22], and not all polymorphism in AMA1 may be relevant to antibody inhibitory activity, which is not accounted for in sequenced-based analyses. Our findings suggest that while large differences in AMA1 sequence are required for vaccine escape, the value of sequence-based analysis for predicting escape from inhibitory antibodies may be limited.

By generating parasites with modified AMA1 alleles, we demonstrated that while the polymorphic C1-L epitope is a significant target of some inhibitory antibodies in GIA, these antibodies only constitute a minor proportion of the total inhibitory antibody repertoire induced by immunisation. W2Mef-AMA1 antibodies targeting the C1-L epitope accounted for 38% of total inhibition, and C1-L also appeared important for HB3. In contrast, C1-L polymorphisms did not have a major effect with the 3D7 allele. This conflicts with previous studies using modified recombinant proteins of 3D7-AMA1 [18], which may differ in the way epitopes are presented compared to native proteins on merozoites. The advantage of using transgenic parasites to dissect specific epitopes or polymorphisms is that AMA1 is presented in its native conformation and context, before and during erythrocyte invasion. The recent phase 2b vaccine trial of 3D7-AMA1 in Mali reported protection against parasites carrying the 3D7 C1 epitope (defined by residues 196, 197, 199, 200, 201, 204, 206, 207) [15]. This indicates that all 8 polymorphic residues in the C1 region may be responsible for modulating strain-specific antibody binding. As the 3D7 and FVO C1-L exchanged in the 3F3 hybrid differed by only 5 residues (aa 196, 197, 200, 204 & 206), it is possible that we did not introduce a sufficiently diversified C1-L to alter inhibitory antibody binding. By testing parasites with different mutations in the C1-L region, our results support the importance of polymorphisms at position 197 for immune escape with the W2Mef allele, but not with the 3D7 allele. The importance of specific polymorphic epitopes and residues may differ between alleles, and the importance of specific epitopes as antibody targets may also differ between naturally-acquired and vaccine-induced responses and between different individuals within a population. Our studies provide an important proof-of-principle for using this novel approach to determine the significance of specific polymorphisms and the transgenic parasite lines we have generated could be valuable to dissect vaccine-induced responses and assess vaccine-escape in future clinical trials.

Prior studies on the inhibitory activity of antibodies induced by immunization with a mixture of three or four different AMA1 alleles also suggest that broadly inhibitory antibodies are generated, although the number of different isolates tested in these studies has been very limited [45]–[46]. These studies also suggested that immunization with a multi-allele mixture enhances the induction of cross-reactive antibodies, which would be beneficial in covering global diversity in AMA1 [45]. Alternative approaches to overcome antigenic diversity of AMA1 have been explored recently. Alanine-mutagenesis of polymorphic C1 residues was used in attempt to reduce the antibody response to highly polymorphic epitopes of AMA1. However, this approach failed to broaden inhibitory-antibody responses towards common epitopes and had a negative effect of reducing the overall inhibitory activity of vaccine-induced antibodies [47]. Another approach being developed in an effort to overcome antigenic diversity is the generation of synthetic AMA1 sequences (DiCo) that aim to represent the majority of AMA1 diversity in three synthetic alleles, giving equal weighting to all polymorphic sites [48]–[49]. Initial reports suggest that this may generate broad growth-inhibitory activity; however, to date, only a small number of different isolates has been tested for inhibition by anti-DiCo antibodies. Our results suggest that the antigenic diversity of AMA1 is not extensive; therefore, a mixture of DiCo antigens, or of naturally-occurring alleles, might be sufficient to overcome antigenic diversity in AMA1. Interestingly, antibodies induced by immunization with a mixture of three DiCo antigens appeared to have lower inhibitory activity than antibodies induced by immunization with a mixture of three naturally-occurring alleles [49].

Our findings support the concept and feasibility of developing a multi-allele AMA1 vaccine for malaria and suggest that the promising strain-specific protective effects shown in the recent phase 2b trial of a mono-allelic vaccine can be broadened using a multi-allele approach. While the four AMA1 alleles used in this study were useful for establishing this concept, further studies are needed to determine the optimum formulation of specific alleles required in a multi-allele vaccine that maximizes coverage in different populations, and to determine concentrations of inhibitory AMA1 antibodies required for protective immunity. The novel approaches developed in this study using transgenic parasites could be valuable to define the importance of specific polymorphic residues and regions for vaccine escape, thereby aiding the selection of specific alleles for vaccine inclusion.
